# Models for Designing Long-Term Care Service Plans and Care Programs for Older People

**DOI:** 10.1155/2013/630239

**Published:** 2013-03-26

**Authors:** Shogo Kato, Satoko Tsuru, Yoshinori Iizuka

**Affiliations:** The University of Tokyo, Healthcare Social System Engineering Laboratory, 7-3-1 Hongo, Bunkyo-ku, Tokyo 113-8656, Japan

## Abstract

The establishment of a system for providing appropriate long-term care services for older people is a national issue in Japan, and it will likely become a worldwide issue in the years to come. Under Japanese Long-term Care Insurance System, long-term care is provided based on long-term care programs, which were designed by care providers on the basis of long-term care service plans, which were designed by care managers. However, defined methodology for designing long-term care service plans and care programs has not been established yet. In this paper, we propose models for designing long-term care service plans and care programs for older people, both by incorporating the technical issues from previous studies and by redesigning the total methodology according to these studies. Our implementation model consists of “Function,” “Knowledge Structure,” and “Action Flow.” In addition, we developed the concrete knowledgebases based on the Knowledge Structure by visualizing, summarizing, and structuring the inherent knowledge of healthcare/welfare professionals. As the results of the workshop and retrospective verification, the adequacy of the models was suggested, while some further issues were pointed. Our models, knowledgebases, and application make it possible to ensure the quality of long-term care for older people.

## 1. Introduction

Japan is known as a “super-aging society [1],” because of long life expectancy and a low birth rate [2]. The population-aging rate (population over 65 years old/total population) in Japan was 23.1% in 2011, which was the highest worldwide [3]. The establishment of a system for providing appropriate long-term care services for older people is a national issue in Japan, and it will likely become a worldwide issue in the years to come [4–6]. In response to this increasing social need for appropriate long-term care services, the Long-term Care Insurance System was instituted in April 2000 in Japan [1]. More than 16.5% of the older people over 65 years of age were using this insurance system in 2008 [7], accounting for about 4,673,000 among the total population of older people (28,317,000).

**Figure 1:**
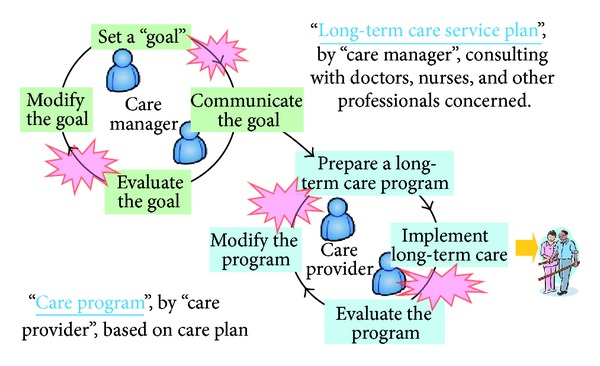
Management system under the Long-term Care Insurance System.

There are two management cycles under this insurance system (Figure 1): one for long-term care service plans, run by care managers, and one for care programs, run by care providers. Long-term care services are based on long-term care service plans, which consist of goals, care services to be provided, estimated care contents, schedules for care services, and so on. Care managers design the long-term care service plans by assessing the condition of an older person and can consult other healthcare professionals concerned—doctors, nurses, and social workers—if needed. Then, each care provider designs a concrete care program based on this long-term care service plan and provides care services according to this care program. Care programs consist of concrete care contents, detailed schedules for care, and so on.

“Excessive care,” “long-term care prevention,” and “dementia care” are mentioned as the three main problems by Muraoka et al. [8] In this study, we focus on the physical care and exclude the mental aspect of care like dementia care and so on. While there are some studies on “long-term care prevention [9–11],” we focus on minimizing or preventing “excessive care.” Excessive care refers to provide higher level of care than the person's actual needs, by corresponding to the person's “demands” or by working for care providers. It serves the care provider's interest and makes the older person comfortable in the short-term. However, it works to the detriment of the older person by reducing the disuse syndrome in the long-term.

In designing long-term care service plans, care managers utilize assessment tools, which are represented by interRAI HC (Resident Assessment Instruments Home Care) [12], and so forth, to analyze problems of the person. However, it is still difficult to design long-term care service plans and care programs that are based on the output of these assessment tools, because the solutions are not associated with the problems in these assessment tools. In the current situation, long-term care service plans are usually designed without determining concrete contents of care, and care programs are usually not designed based on the specific condition of each older person. As a result, the quality of long-term care service plans, long-term care programs, and therefore the quality of long-term care for older people has been dependent upon the particular experience or attitude of a care manager or care provider. Thus, it is necessary to establish appropriate processes for designing long-term care service plans and care programs according to the specific condition of each older person.

With respect to the background provided above, Kato et al. [13] proposed a process model for determining elderly care according to ADL (activities of daily living). This model would help care managers determine the concrete contents of care suited to an elderly person's ability to perform each ADL. In addition, Kato et al. [14] developed the necessary knowledge contents for ADL items that would be used in the process model for determining elderly care. Using this model and the knowledge contents, we can more precisely determine concrete contents of care suited to an elderly person's ability to perform each ADL. 

However, these individual studies have not managed to establish a complete methodology for designing long-term care service plans and care programs, for two reasons. First, these studies had a limited scope and did not examine the full range of issues in elderly care. Second, they had a few technical issues: the model could not be adapted to suit individual environmental needs; it was difficult to determine which care services should be included according to the output of the model, because care services were not associated with individual care options, and so on.

In this study, we propose a logical model and implementation model for designing a long-term care service plan for older people, both by incorporating the technical issues from previous studies and by redesigning the total methodology according to these studies [13, 14]. We interpreted the design of a long-term care service plan and care program as the design of practicable measures that can satisfy the needs of an older person, which can be considered as a “needs-seeds problem”; accordingly, we have designed an appropriate model. We resolved the above two problems noted in the previous studies and endeavored to help care managers and care providers visualize and develop more structured thinking processes. This study is limited to long-term care in terms of ADL items, because we consider ADL to be one of the most fundamental parts of a person's life.

## 2. Materials and Methods

### 2.1. Designing the Models

#### 2.1.1. Core Concept

**Figure 2:**
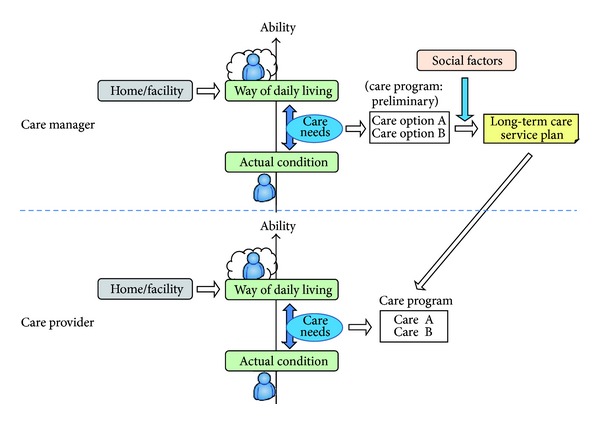
Core concept.

We interpreted the processes of designing a long-term care service plan and care program as a process of designing practicable measures to satisfy the care needs of an older person. Long-term care is required when an older person wants to realize a specific way of daily living, depending on the person's current ability and the conditions of their home/care facility. That is, when an older person wants to realize a specific way of daily living, he or she requires various physical and mental abilities, and acquiring such abilities depends on the conditions of his or her home/care facility. If the older person has sufficient ability, he or she might be able to realize the way of daily living by him or herself. However, if he or she does not have sufficient ability, he or she would not be able to realize this way of daily living independently and thus would require assistance. Some care services are planned to provide such assistance. From this fundamental concept, we propose the core concept for designing a long-term care service plan and care program, as shown in Figure 2.

This process of designing a long-term service plan and care program involves different duties for care managers and care providers. These duties share many elements and can be categorized as the following six “functions.” Care managers are responsible for Functions 1–6, while care providers perform Functions 1–5 using the guidelines of the long-term care service plan.

 Function 1: Assess the person's ability
 Function 2: Assess home/care facility condition
 Function 3: Assume a hypothetical way of daily living
 Function 4: Identify care needs
 Function 5: Design care program
 Function 6: Design long-term care service plan.

#### 2.1.2. Designing the Logical Model

The first step of this study was limiting the scope of older people's ADLs to focus on six items: moving, dressing, eating, grooming, urinating, and bathing. We did this according to previous literature [15–17].

In general, there are multiple ways to perform each ADL. To express the variety of ways, an ADL needs to be broken down into individual “element actions.” At that point, each ADL can be expressed as a “realization pattern,” which is a combination of multiple element actions. To evaluate whether the older person can achieve each element action independently, “ability elements” are introduced as indicators to assess the person's condition. The actual condition of the person can be quantitatively expressed as a score for each ability element, which is referred to as “actual ability.” The ability required for the person to perform an element action is referred to as the “required ability for element action.” Required ability also depends on some conditions of the residential area where the person is living, which are referred to as “home/facility conditions.” Then, for each ability element, a gap is identified by comparing actual ability with required ability for each element action; the gaps between actual and required ability represent the needs to be met by long-term care.

Generally, there are multiple care options to satisfy these identified care needs. The required ability for a person to perform a certain element action called for by a given care option is referred to as “required ability for care.” Each care option is evaluated on whether it can satisfy the person's care needs, by comparing the actual ability with the required ability for care. In addition, multiple care services might be able to provide the required care. The long-term care service plan should be designed by considering social factors such as financial problems or family issues. Thus, according to the above consideration, we designed the logical model.

#### 2.1.3. Designing the Implementation Model

As described below, we suggest that each function in the logical model should consist of multiple information conversions, and factors to be considered, like element action, ability element, care, and so forth, would be deployed into a considerable number. Thus, we needed to design an implementation model, by further specifying the functions, structuring the information flow, and developing the knowledgebases according to structured knowledge from experts. In this study, we created three concrete components for this implementation model: “Function,” “Knowledge Structure,” and “Action Flow.”

#### 2.1.4. Developing the Knowledgebases and Application

We developed concrete knowledgebases, based on the Knowledge Structure, in collaboration with care managers, doctors, nurses, and social workers in the Ohme area. In the development process, we applied the knowledge contents for twelve actual cases that were being handled by concerned professionals to ensure that the knowledgebases were accurate. 

In addition, we developed a specific computer application using Microsoft Excel and Visual Basic for Applications (VBA), known as the “prototype system,” in order to carry out the methodology specified in the implementation model.

### 2.2. Methods for Verification of the Model

In an attempt to perform initial validation of the proposed model, one of the authors (Kato) held a workshop together with five care managers (two newly recruited, two experienced, and one advising) to design care programs for some older people at the one of care facilities in Tokyo.

#### 2.2.1. Preparing the Cases

 In this workshop, Kato and the care managers developed care programs for two actual cases. The actual cases were in the charge of the advising care manager, and neither the other care managers nor Kato had prior information about these cases. One was the case of higher care-need level (level 5), and another was the case of lower care-need level (level 1).

In this workshop, we limited the ADL item of urinating. care for eating, urinating, and bathing are known as the “three major care” in Japan, and the forms of care for urinating changes a lot based on the person's condition in these three, so we considered that focusing on urinating was appropriate as the first step.

#### 2.2.2. Grouping

Kato and the four care managers were divided into three groups (A, B, and C). A newly recruited care manager and an experienced care manager were included in both Groups A and B, aiming to reduce the variation between groups. Kato was the only member of Group C.

 Group A did not use the model and carried out all the steps manually, while Group B used a format sheet on Excel, that reflected the implementation model (Function, Knowledge Structure, and Action Flow), but without knowledgebases. It means that the Group B designed care programs, by developing required knowledgebases by themselves according to the format sheet. This group was received the instruction of the use of the format sheet before starting the workshop. Group C used the prototype system containing the knowledgebases. It means that the output of Group C would not depend on the specific professional ability of the group member, but would depend on the prototype system.

#### 2.2.3. Designing Care Programs

Every participant independently designed care programs for two cases, after receiving explanations for each case from the advising care manager together. The amounts of time required were approximately 10 to 20 minutes for each case per participant.

#### 2.2.4. Preparing the Reference Programs

It was difficult to evaluate the output of each group by directly comparing them, because both the expression form and granularity of the description were quite different. We prepared the reference program by modifying the output of the prototype system based on the opinion of the advising care manager, to make it possible to compare the output of each group.

#### 2.2.5. Evaluating the Output of Each Group

As the evaluation indicators of care program, we adopted three indicators: completeness, definiteness, and accuracy [13, 14].

These factors were assessed through discussions among all the participants of the workshop by comparing the outputs of the three groups with a reference program. 

### 2.3. Methods for Verification of the Knowledgebases and Application

We conducted a retrospective verification of the knowledgebases and application with 7 healthcare/welfare professionals by using the prototype system in Tokyo.

#### 2.3.1. Selecting Cases

We applied the prototype system to total 50 actual cases who were in the charge of seven experienced (each having more than five years) healthcare/welfare professionals (two nurses: N1 and N3; two social workers: S4 and S5; and three care managers: M1, M3, and M4).

We asked the participating healthcare/welfare professionals to select various cases, that is, various levels of care-needs, age, causative diseases, and so on, for application to confirm the validity of our proposal.

#### 2.3.2. Evaluating the Knowledgebases and Application

As the evaluation indicators of care program, we adopted “effectiveness to provide excessive care” in addition to the previous three indicators as listed in Section 2.2 (completeness, definiteness, and accuracy). 

These 4 indicators were used as a basis to create the following 4 questionnaires, and we asked the participating healthcare/welfare professionals to evaluate the following four points, for five ADL items: dressing, eating, grooming, urinating, and bathing. Eventually, we evaluated these indicators for a total of 250 ADL items.

 R1: Does the judgment made on each element action and care option in the output agree with the real life situation of the case, as understood by the professionals in charge of that case? (Characteristic 3: accuracy)
 R2: Are there ambiguous or difficult to understand expressions in the output of the prototype system? (Characteristic 2: clarity)
 R3-1: Did the real life situations follow the system-derived plan? (Characteristic 1: completeness)
 R3-2: Are there any element actions or care options lacking from the output? (Characteristic 1: completeness)
 R4: Was there a more appropriate realization pattern from a longevity perspective? (Characteristic 4: effectiveness)

## 3. Results and Discussion

### 3.1. Results: Logical Model

**Figure 3:**
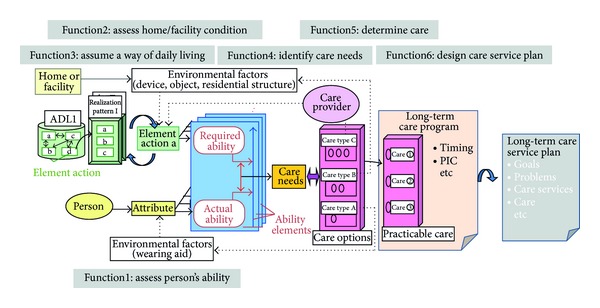
Logical model.

The designed logical model is shown in Figure 3. The logical model is a methodology for designing a long-term care service plan and accompanying care programs, by showing all the elements that need to be considered in this process as well as the relationships between them. The six functions in the logical model are described as follows.

#### 3.1.1. Function 1: Assess the Person's Ability

We evaluate the actual condition of an older person. An “ability element” is a scale used to assess a person's condition, such as the “ability to see,” and “ability to keep standing position.” The actual condition of a patient can be quantitatively expressed as a score for each ability element, which is referred to as “actual ability.” If the person used any wearable supporting devices, we must assess the ability of the person separately for instances where devices were worn and for those where they were not worn.

#### 3.1.2. Function 2: Assess Home/Care Facility Condition

We assess the condition of the person's home/care facility, because it is necessary to consider the functional design and residential structure of home/care facility where the person is living, as well as the available human and physical resources, among other aspects. 

#### 3.1.3. Function 3: Assume a Hypothetical Way of Daily Living

We assume an example way of daily living, which that person might want to realize. We do this by assuming how the person currently performs each ADL. In general, there are multiple ways to perform each ADL. To express the variety of ways in which each ADL item can be achieved, each ADL must be divided into more basic “element actions.” Subsequently, each ADL is expressed as a “realization pattern,” which is a combination of multiple element actions based on the condition of that person's home/care facility. 

It is not always the best way to determine simply the way of daily living which the older person or families “want,” because there are comprehension gradients between care managers and the older person/families. We need to assume a way of daily living here, based on the results of Function 1 and 2, by considering the hopes of the person/families.

#### 3.1.4. Function 4: Identify Care Needs

Here, we identify an elderly person's needs for long-term care. The ability required for a patient to perform an ADL for the way of daily living that person is seeking is referred to as “required ability for element action.” For each element action in the realization pattern, ability gaps are identified by comparing the actual ability with the required ability for element action. These ability gaps represent that person's care needs. 

#### 3.1.5. Function 5: Design Care Program (Preliminary)

Here, we determine a feasible method for satisfying the identified care needs. These methods are classified into two types. One method is “decreasing the required ability for action,” which can be achieved by improving the environmental conditions, using supporting devices, and providing assistance. The other method is “improving actual ability,” which can be achieved by rehabilitation training or by the use of wearable supportive devices (e.g., eyeglasses or acoustic aids).

The practicable measures for each older person are selected from the multiple long-term care options capable of filling the ability gaps that were identified for each element action, by comparing the actual ability with the required ability for care.

Then, we designed a care program by determining the frequency and timeline for the implementation of long-term care and the person in charge (family or service provider), by considering the hopes of the person/families.

#### 3.1.6. Function 6: Design Long-Term Care Service Plan

Finally, we design a long-term care service plan based on the preliminary care program, by considering social factors like financial problems or family issues and hopes of the person/families.

### 3.2. Results: Implementation Model

**Figure 4:**
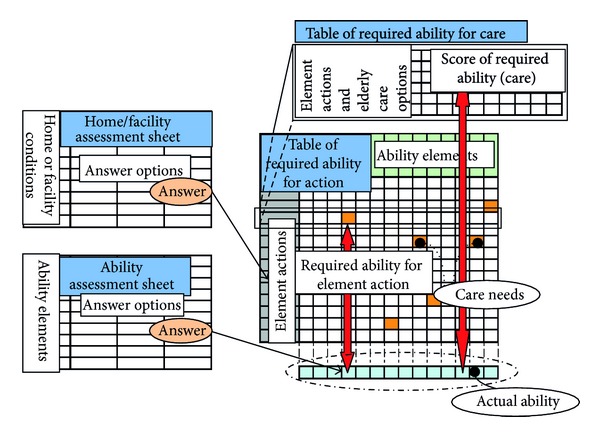
Knowledge Structure.

**Figure 5:**
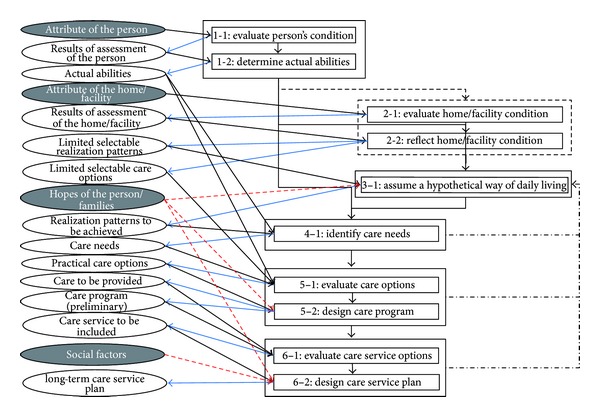
Action Flow.

For the Implementation Model, Table 1 shows the “Function” component; Figure 4 shows the “Knowledge Structure” component; and Figure 5 shows the “Action Flow” component. The Function component computes the various information conversions that can be used to find a final solution; Knowledge Structure indicates the structure of knowledge used to implement the Function; finally, Action Flow represents the procedure and flow of information during implementation of the model's methodology. 

As shown in Figure 4, the knowledge contents based on this Knowledge Structure were converted into four knowledgebases: “Ability Assessment Sheet,” “Home/Facility Assessment Sheet,” “Table of Required Ability for Element Action,” and “Table of Required Ability for Care.” 

**Figure 6:**
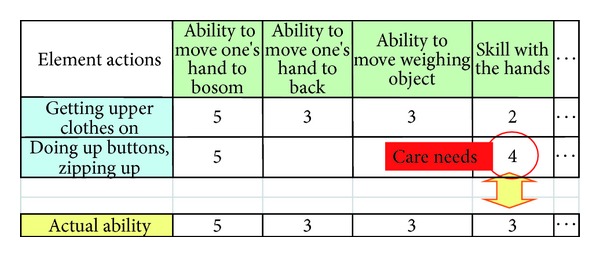
Example of Function 3–1 (partial).

For example, Figure 6 shows the parts of Function 3–1, by using the table of required ability for action. In this example is expressing the parts of results of 3–1 for “dressing.” In this realization pattern, element actions like “getting upper clothes on,” “doing up buttons, zipping up,” and so forth and ability elements like “ability to move one's hand to bosom,” “ability to move one's hand to back,” “ability to move weighing object,” “skill with the hand,” and so forth are included. Care need is identified for the element action “doing up buttons, zipping up” on ability element “skill with hand,” because the required ability for this element action on this ability element is set as 4, which is higher than the actual ability assessed as 3.

### 3.3. Results: Knowledgebases and Application of the Model

We developed the knowledgebases within the scope of the implementation model. For example, the Table of Required Ability for Element Actions contains 125 element actions and an organized Required Ability list, which was created using 32 Ability Elements.

In addition, we developed a specific application using Excel and VBA. The methodology specified by the implementation model can be carried out using this application, referred to as the “prototype system.” The prototype system carries out Functions 2~5 after the user has performed Function 1. Therefore, the user can practice functions 5–2 to 6–2, with supporting information from the prototype system (functions are shown in Table 1).

### 3.4. Results: Verification of the Model

The results of Section 2.2.5 are summarized in Table 2. In Table 2, three evaluation indicators (completeness, definiteness, and accuracy) are arranged and are divided into subindicators in vertical row, and care managers are arranged for each case in the horizontal row. Two realization patterns were assumed for the second case. In Table 2, numbers of differences between each output from the reference program are described in each cell. The smaller these numbers are, the better the quality of each output is.

For example, for “shortage of cares” in each output for the first case, there were 8 shortages of cares in the output of A1, 4 in that of A2, 4 in that of B1, 1 in that of B2, 2 in that of C (by knowledgebases before modification), and 0 in that of C (by modified knowledgebases). For this subindicator, we can evaluate that the quality of each output is in the following order: C∗ > B2 > C > B1 = A2 > A1.

According to the three evaluation indicators, we found that the overall outputs of Group C were the best, followed by those of Group B and then Group A. The outputs of Group B, which used the format representing the Function, Knowledge Structure, and Action Flow, were found to be better than those of Group A, which did not use the logical model at all. Further, the outputs of Group C, which used the entire implementation system, were better than the outputs of Group B. 

When examining some parts of the outputs, however, the ranking order mentioned above (Group C, Group B, and then Group A) was not necessarily valid. A comparison between Groups A and B revealed that some of the outputs of Group A were better than the corresponding outputs of Group B. The overall outputs of Group C were of a higher quality because Group C used the knowledgebase built using the implementation system. Group C, however, failed to present some outputs which Groups A and B were able to produce. 

When we compared the outputs of the newly recruited care manager (expressed as “fresh” in Table 2) and the experienced care manager from the same group, we found that some outputs of the newly recruited care manager were better than the corresponding outputs of the experienced care manager.

### 3.5. Results: Verification of the Knowledgebases and Application

The results of Section 2.3.2 are shown below.

*(R1-Accuracy): Does the Judgment Made about Each Element Action and Care Option in the Output Agree with the Real Life Situation of the Case, As Understood by the Professionals in Charge of That Case? *There were a total of 22 misjudgments of element actions or care options by the prototype system when applied to a total of 50 cases and 250 ADL items. Each ADL item was expressed as realization patterns made by approximately 5–15 element actions. The breakdown of mistakes found in the output judgments is as follows:

 M1 (care manager): 13 (within five cases)
 N1 (nurse): 8 (within eight cases)
 M3 (care manager): 1 (within six cases).

We consider that the rate of mistakes in the output of the prototype system is sufficiently low, and it is possible to reduce the rate further by improving the knowledgebases continuously in the future. We can say that the knowledgebases and the application have enough accuracy from these results.

*(R2-Definiteness): Are There Ambiguous or Difficult to Understand Expressions in the Output of the Prototype System?* There are no ambiguous and unclear expressions pointed in the output when applied to a total of 50 cases. We can say that the knowledgebases and the application have enough definiteness from this result.

*(R3-Completeness): Did the Real Life Situations Follow the System-Derived Plan? (R3-1), Are There any Element Actions or Care Options Lacking from the Output? (R3-2)* Real-life situations were found among the possible realization patterns for 249 of the total 250 ADL items without shortage of the realization patterns. While some actions for the preparation/management of environmental factors were indicated as absent in the realization pattern, these factors were judged to be unnecessary as the standard output at this time through discussion among participants of the workshop.

We can say that the knowledgebases and the application have enough completeness of realization patterns, element actions, and care options, while some further issues were suggested.

*(R4-Effectiveness to Prevent Excessive Care): Was There a More Appropriate Realization Pattern from a Longevity Perspective?* For 125 of the 250 ADL items, participating professionals found a realization pattern considered to be more appropriate than the current one utilized by the case, from the perspective to prevent excessive care. For example, there are some cases, where older people were eating with total assistance by care provider, in spite of the fact that they could eat with only partial assistance.

It is usually difficult for care manager or care provider to lower the level of assistance without some kind of evidence. We can say that our application with the knowledgebase has the potential to be used as such evidence and will contribute to minimize excessive care.

### 3.6. Discussion

#### 3.6.1. Initial Validation of the Model

We consider the logical model to be valid, because of the overall results of the workshop—which indicated that Group C had the best procedure, followed by Group B and then Group A—as well as the results of the application to actual cases. 

In the workshop, the care managers of Group B were able to effectively utilize their experience and expertise because they used the format reflecting the implementation model. As a result, Group B was able to generate higher quality outputs than Group A. Group A performed better than Group B on several areas; however, we concluded that the experience and expertise of the therapists from Group A was superior in those areas to that of the therapists from Group B. The outputs of Group C were of a higher quality because its sole member used the knowledgebases that we designed in the initial phase of our study. However, Group C failed to present some outputs that Groups A and B were able to produce. We believe this is because the knowledgebases used in this workshop was developed with only a few professionals, and there is room to further improve the scope of the knowledgebases

The workshop was held with a minimum size of cases and care managers. While we consider that the results were enough as the initial validation of the model, we will need more tests in the future through expanded size of cases and care managers.

#### 3.6.2. Validation of the Knowledgebases and Application

In the application to actual cases, the adequacy of the knowledgebases and application was confirmed on four evaluation indicators: completeness, definiteness, accuracy, and effectiveness to prevent excessive care. According to the results in Section 3.5, it was suggested that our knowledgebases and application would have the potential to be supporting evidence for the appropriate level of care and would contribute to minimize or prevent excessive care.

It was also suggested that the quality of the knowledgebases needed to be improved continuously for future studies. In this study, we have developed the knowledgebases with a small number of care managers in an area in Japan. Therefore it is possible that the scope of the knowledge is dominated by the characteristics of the region. We will need more test widely in the future in the various areas in Japan.

#### 3.6.3. Significance of the Model

We believe that our model will be able to meet the social needs of Japan and perhaps might eventually meet those needs across the world, as there is currently no existing standardized methodology for designing long-term care service plans. This is due in part to the characteristics and structure of the essential problem: the “needs-seeds” problem. We interpreted the designing of a long-term care service plan and care program as designing practicable measures to satisfy the care needs of an older person; in our model, we did this by converting a way of daily living into component elements and the required ability for those elements, and therefore relating the care options to the ability gaps.

We developed concrete knowledgebases by using the Knowledge Structure that we designed. However, the case study results suggested that the Knowledge Structure itself was adequate. In addition, it is more important that we develop an adequate body of knowledge (BOK) in this area and make it accessible and improvable, as those are essential factors in the establishment of the methodology as a sociotechnical system [18, 19].

Our model is designed specifically for long-term care. By generalizing accordingly, however, this model can serve as the basis for a common model that can be used to design practicable measures to satisfy needs expressed as gaps between actual conditions and assumed conditions. We plan to attempt to apply this model to various issues in elderly care and then improve it to the point that it could perhaps handle each individual issue.

In addition, our model includes the perspective of “designing tool for ways of daily living,” and it can be available for various professionals and people other than care managers and care providers. While we consider that any special skills are not strongly required for the use of our model, professionals like nurses, social workers, therapist, and so forth, who have a skill for evaluation of daily living, will benefit as they can use our model to be more effective and efficient. For people without such a skill, our model is expected to work as an educational tool for evaluation of daliy living.

#### 3.6.4. Future Issues

We need further consideration on the acknowledgment-of-ability elements. Further study on these abilities (i.e., individualized functions) is also under way in this specialized field. In the future, we will endeavor to develop proper indicators (ability elements) and identify the relationships between acknowledgement-of-ability elements and element actions. Once this issue is resolved, we believe that it will be possible to precisely estimate the required time for providing care to a target client, as well as to conduct a benchmark test for the current Long-Term Care Insurance System.

In this study, we developed knowledgebases focusing on ADL and outlined a set of functions (1–5) that were covered by these knowledgebases. However, we need to extend the scope of these knowledgebases to include elements other than ADL and more fully cover Function 6 in the future.

In addition, we developed the knowledgebases by structuring the technical knowledge obtained from a specific group of healthcare/welfare professionals. Therefore, the knowledgebases could partially depend on their specific knowledge. In addition, the knowledge bases could depend upon cultural and social values. In the future, we will need to consider these issues in other groups of healthcare/welfare professionals and make further verifications to develop more standardized knowledgebases.

#### 3.6.5. Integration of the Proposed Model into PCAPS-IMT

To ensure quality healthcare, Iizuka et al. [20] proposed a system known as the PCAPS-IMT (patient condition adaptive path system). The distinguishing feature of this system is that it adapts to the individual patient's particular conditions. PCAPS-IMT consists of two tools. The clinical process chart encompasses the overall flow of clinical judgments and treatments that can be considered for a type of disease, consisting of clinical unit processes, and the unit sheet specifies a set of treatments, tests, observations, and other clinical treatments to be conducted in a unit clinical process to manage the total activities by the clinical team.

To obtain a satisfactory performance using this system, it is necessary to correctly understand the patient's specific conditions. Therefore, we consider that it is possible to integrate the process model for determining elderly care into the PCAPS-IMT as an evaluation model for patient scenarios. In particular, we consider it to be applicable during periods of convalescence, where the degree of dependence upon nursing is critical. Accordingly, we will consider applying our model in such situations.

## 4. Conclusions

In this study, we have developed a model that can be used to design appropriate long-term care service plans and accompanying care programs. Furthermore, through the implementation model we also established knowledgebases for long-term elderly care and a prototype system using Excel and VBA. By using the proposed model, it is possible to efficiently and effectively design a long-term care service plan and care program suited to the way of daily living, which an older person wants to realize.

To promote sustainable growth in society, it is essential to meet the social needs associated with the long-term care of the elderly. This study is expected to contribute considerably in the efforts to ensure long-term care for the elderly and improvement of the quality of such care.

## Figures and Tables

**Table 1:** Function.

Name			Input	Activity	Output	Required knowledgebase
1: assess the person's ability	1-1	1-1: evaluate person's condition	*Attribute of the person *	Evaluate person's condition	Results of assessment of the person	Ability assessment sheet
	1-2	1-2: determine actual abilities	Results of assessment	Determine actual ability of the person on the basis of the results of 1-1	Actual abilities	(Logic for determining actual abilities: included in ability assessment sheet)

2: assess home/facility condition	2-1	2-1: evaluate home/facility condition	*Attribute of the home/facility *	Evaluate environmental condition of the home/facility, where the person will live	Results of assessment of the home/facility	Home/facility assessment sheet
	2-2	2-2: reflect home/facility condition	Results of assessment of the home/facility	Limit selectable realization pattern and care option on the basis of the results of 2-1	(i) Limited selectable realization patterns (ii) Limited selectable care options	(Table of limitation to realization pattern and care option: included in the home/facility assessment sheet)

3: assume a hypothetical way of daily living	3–1	3–1: create a hypothetical mode of life	(i) Limited selectable realization patterns (ii) *Hopes of the person/families *	Set (multiple) realization patterns for the person on the basis of the results of 2-2, considering the hopes of the person/families	Realization patterns to be achieved	(List of Realization Pattern: included in the table of required ability for element action)

4: identify care needs	4–1	4–1: identify care needs	(i) Realization patterns to be achieved (ii) Actual abilities	Identify person's care needs as gaps between required ability for (multiple) realization patterns set in 3–1 and actual abilities determined in 1-2	Care needs	Table of required ability for element action

5: design care program (preliminary)	5–1	5–1: evaluate care options	(i) Care needs (ii) Limited selectable care options (iii) Actual abilities	Evaluate practicability of care options to meet the person's care needs identified in 4–1 and actual abilities determined in 1-2	Practicable care options	Table of required ability for care
	5–2	5–2: design care program	(i) practicable care options (ii) *hopes of the person/families *	Determine care to be provided and design care program from the care options found practicable in 5–1, considering the hopes of the person/families	(i) Care to be provided (ii) Care program	—

6: design long-term care service plan	6–1	6–1: evaluate care service options	Care to be provided	Determine care services to implement elderly care selected in 5–2	Care services to be included	Table of care service option
	6–2	6–2: design care service plan	(i) care services to be included (ii) *hopes of the person/families* (iii) *social factors *	Design a long-term care service plan on the basis of the results of 6–1, considering the hopes of the person/families	Long-term care program	(Design item (PIC, Timeline, etc.))

**Table 2:** Results of the verification.

		Case, care manager														
		Case I (Normal)					Case II (Wheelchair)					Case II (Portable Toilet)				
	Evaluations	A		B			A		B			A		B		
		A1 (fresh)	A2 (experienced)	B1 (fresh)	B2 (experienced)	C	A1 (fresh)	A2 (experienced)	B1 (fresh)	B2 (experienced)	C	A1 (fresh)	A2 (experienced)	B1 (fresh)	B2 (experienced)	C
Completeness	Shortage of element actions	9	5	4	4	1 (0)*			4	5	1 (0)*	7	6	3	3	1 (0)*
	Shortage of cares	8	4	4	1	2 (0)*	—	—	4	3	2 (0)*	8	8	4	2	2 (0)*

Definiteness	Ambiguous expressions	1	2	0	0	0	—	—	0	0	0	1	1	0	0	0

Accuracy	Misjudgment for element actions (misjudged as NG)	2	2	2	0	2 (0)*			0	0	0	0	0	0	0	0
	Misjudgment for element actions (misjudged as OK)	1	2	1	0	0			0	0	0	0	0	0	0	0
	Misjudgment for care (misjudged as OK)	2	2	2	0	0	—	—	0	0	0	0	0	0	0	0
	Misjudgment for care (misjudged as NG)	3	6	3	0	1 (0)*			0	0	2 (0)*	0	0	0	0	2 (0)*

*Number by Knowledgebases before modification (number by modified Knowledgebases).
